# Bilateral Symmetry in the Aesthetic Area Achieved by Digital Smile Design on 3D Virtual Patient and Conventional Diagnostic Wax-Up—A Comparative Study

**DOI:** 10.3390/dj12120373

**Published:** 2024-11-21

**Authors:** Maria Hristozova, Mariya Dimitrova, Stefan Zlatev

**Affiliations:** 1 Department of Prosthetic Dentistry, Faculty of Dental Medicine, Medical University of Plovdiv, 4002 Plovdiv, Bulgaria; stefan.zlatev@mu-plovdiv.bg; 2 CAD/CAM Center of Dental Medicine, Research Institute, Medical University of Plovdiv, 4000 Plovdiv, Bulgaria

**Keywords:** dental symmetry, smile design, digital dentistry, digital diagnostic waxing, dental aesthetic

## Abstract

**Background**: Digital diagnostic waxing is a contemporary alternative to the conventional wax-up method. This study aims to evaluate the impact of both techniques on the perceived frontal symmetry in aesthetic treatment planning. Dental symmetry significantly influences smile perception and, consequently, the acceptance of treatment outcomes, highlighting its clinical importance in restorative dentistry. **Materials and Methods**: A total of 100 teeth were measured, with 50 (*n* = 50) waxed up using traditional modeling techniques and 50 using a face-guided digital approach. The study involved ten patients requiring fixed restorations in the aesthetic zone. Both digital and conventional wax-ups were performed for each participant. Gypsum models with wax-ups were digitized and superimposed onto the digital diagnostic design using 3Shape Dental Designer Studio software, Version 2023 (3Shape, Copenhagen, Denmark). Screenshots of the frontal view were captured, and the width of each morphologically altered tooth was measured using ImageJ software Version 1.54 (National Institutes of Health, Bethesda, MD, USA). **Results**: The results indicated no statistically significant difference in symmetry between the right and left sides achieved by the two diagnostic approaches (t-value = −1.89, *p*-value = 0.07). The perceived symmetry of morphologically modified frontal teeth, as achieved by digital and conventional waxing, was found to be comparable. **Conclusions**: Digital diagnostic planning is validated as a reliable alternative to the conventional wax-up method, offering comparable accuracy in achieving dental symmetry while potentially enhancing efficiency and precision in the aesthetic treatment planning process. This result underscores the potential of digital technologies to streamline clinical workflows and improve patient outcomes. Clinically, achieving symmetry in the aesthetic zone is crucial for patient satisfaction and acceptance of restorative procedures, emphasizing the need for continued integration of digital tools in dental practice.

## 1. Introduction

Modern dentistry has evolved to encompass not only the morphological and functional restoration of dentition but also the enhancement of dental aesthetics and the reversal of visual signs of aging [1,2,3,4,5]. This shift reflects a growing recognition of aesthetics’s integral role in a patient’s overall well-being [6]. The smile, in particular, is universally regarded as the most significant physical feature influencing perceptions of attractiveness and self-esteem across both genders [7,8,9]. Notably, dental aesthetics is not a new trend; its roots can be traced back to ancient times when the appearance of teeth was valued alongside health. Across cultures and centuries, the smile has been universally regarded as a significant physical feature influencing perceptions of attractiveness and self-esteem [10]. In experimental research conducted by Beall A.E., it was convincingly demonstrated that teeth profoundly impact how one’s personality and appearance are perceived by others [8]. This underscores the importance of dental aesthetics, which are closely associated with positive social interactions, heightened self-confidence, and enhanced psycho-social well-being and oral health-related quality of life [9,10].

Symmetry is fundamental in dental aesthetics, as it is closely associated with perceptions of beauty and harmony. Beyond the smile and facial structure, achieving precise symmetry in individual teeth—especially in the central incisors—is essential, as these teeth serve as focal points in the aesthetic composition of the smile. The golden ratio, representing visually pleasing proportions, has been extensively utilized in dentistry to achieve optimal balance not only between facial and dental features but also within the dentition itself, underscoring the importance of proportional alignment in anterior teeth [11,12,13]. The influence of dental aesthetics extends well beyond mere appearance; changes in visible teeth can trigger psychological responses that significantly affect how patients view and accept prosthetic solutions. This acceptance is crucial, even when treatments are performed with impeccable technical and functional accuracy, highlighting the substantial impact of aesthetics on patient satisfaction [14,15]. In light of these insights, dental treatment goals have evolved considerably. Contemporary dental practices focus on preventing and treating conditions related to the oral cavity’s hard and soft tissues and enhancing overall well-being and quality of life [16,17]. This improvement is pursued by aiming for exceptional aesthetic results, with a strong emphasis on minimally invasive methods that prioritize the preservation of the natural dentition [18,19,20].

The shift toward aesthetics-driven dental care mirrors a broader trend in healthcare that values patient-centered outcomes, where success is measured not only by clinical standards but also by improvements in patients’ quality of life [21]. Advanced technologies and materials in aesthetic dentistry are now essential for meeting patients’ functional and emotional needs, aligning with healthcare’s goal of enhancing overall well-being, as dental aesthetics boost self-image, confidence, and social interactions, thereby contributing to a better quality of life [22,23]. A prominent example is the facially driven digital diagnostic wax-up, which, using facial measurements, predicts aesthetic outcomes more accurately than traditional methods and streamlines the planning process [24]. This digital wax-up reduces costs by minimizing physical materials and adjustments, offers easy storage and retrieval, and is reversible, allowing for changes without physical alterations [4,25]. By increasing precision and efficiency, digital tools ultimately improve treatment outcomes and patient satisfaction, enabling dental professionals to deliver more predictable, optimized results [17].

Restoring anterior teeth with changes in display, shape, alignment, or size can greatly impact a patient’s self-esteem and perception of success [24]. To ensure patient satisfaction, aesthetic prosthodontic treatments should include previsualization of the changes, often achieved through a diagnostic wax-up [25]. This wax-up simulates the anticipated outcome, enhances clinician/patient communication, and serves as a foundation for creating mock-ups, guiding treatment, and fabricating provisional restorations, leading to more predictable results [26,27]. Using such diagnostic tools helps manage patient expectations and aligns the final results with desired aesthetic and functional goals [28,29].

Digital technology’s emergence has transformed dentistry, allowing for complete aesthetic analysis and treatment planning within a virtual setting [16]. This modern method includes several essential steps: initially, the patient’s facial features and dentition are captured using advanced imaging techniques [5]. This digital information is combined by overlaying different file formats, creating a detailed 3D simulation of the patient’s dentition in a static virtual model known as the “virtual patient model” [11]. While digital and conventional wax-ups provide a solid foundation for aesthetic planning, incorporating a three-dimensional (3D) analysis of symmetry could offer deeper insights into facial and dental harmony. Unlike traditional two-dimensional approaches, 3D analysis accounts for depth, contour, and volumetric relationships between teeth, especially the central incisors, which play a critical role in overall aesthetic balance [24]. This added dimension enables clinicians to detect subtle asymmetries that might be overlooked in two-dimensional views, enhancing precision in designing restorations that harmonize with the patient’s unique anatomy. Integrating 3D symmetry analysis within digital workflows improves treatment predictability and helps achieve restorations that better mimic natural aesthetics, contributing to greater patient satisfaction.

The impact of digital prosthodontic planning on dental aesthetics was thoroughly evaluated by Abduo et al. [15]. Abduo’s study found that while digital and conventional waxing protocols generally yield comparable outcomes, the digital approach needs further validation to establish its reliability and effectiveness fully [28]. Similarly, in a more recent investigation, Chisnoiu et al. compared conventional and digital smile design and treatment planning methodologies [29]. Chisnoiu’s research indicated that traditional analog methods received slightly higher average ratings than digital techniques, suggesting that while digital methods advance, they may still lag behind their conventional counterparts in certain aspects of aesthetic evaluation [30,31].

The primary objective of this study is to compare the bilateral symmetry of the frontal dentition achieved through digital smile design on a 3D virtual patient with that obtained through conventional diagnostic wax-ups. The null hypothesis for this study is that the type of diagnostic wax-up employed (digital versus conventional) will not significantly impact the achieved bilateral symmetry in the transverse plane of the mirroring teeth from positions 13 to 23. By evaluating this variable—symmetry—the study aims to determine whether digital and conventional methods produce comparable aesthetic outcomes. The findings could offer insights into the effectiveness and accuracy of digital technologies in achieving aesthetically pleasing and symmetrical results, potentially influencing future practices in dental prosthodontics and smile design.

## 2. Materials and Methods

### 2.1. Study Design

This study utilized a controlled trial design to evaluate the effectiveness of digital and conventional wax-up modeling techniques in achieving bilateral symmetry in anterior dentition. The study examined 10 patients, resulting in a total of 50 teeth that were modeled using both approaches. The study was conducted by three researchers who were involved in the design, execution, and analysis phases.

### 2.2. Participants

Ten patients were selected based on specific inclusion criteria: each required improvements in the dental aesthetics of at least one anterior tooth or modifications to their visible maxillary dentition, as dictated by an overarching prosthetic rehabilitation treatment plan (Table 1). These inclusion criteria ensured that the patients would benefit from the aesthetic enhancements being evaluated. Ethical approval for the research was obtained from the Scientific Ethics Committee of the Medical University of Plovdiv, confirming compliance with ethical standards for human research (Protocol No.4/4 May 2023).

The treatment plan for eight of the patients involved prosthetic rehabilitation with metal–ceramic crowns and bridges. One patient (Patient No.1) received combined treatment with all-ceramic restorations, including a three-unit bridge and three veneers. The type of final restoration chosen does not affect the study, as the focus is on comparing the perceived symmetry achieved through a facially guided aesthetic treatment plan and conventional diagnostic waxing.

### 2.3. Interventions

**Figure 1:**
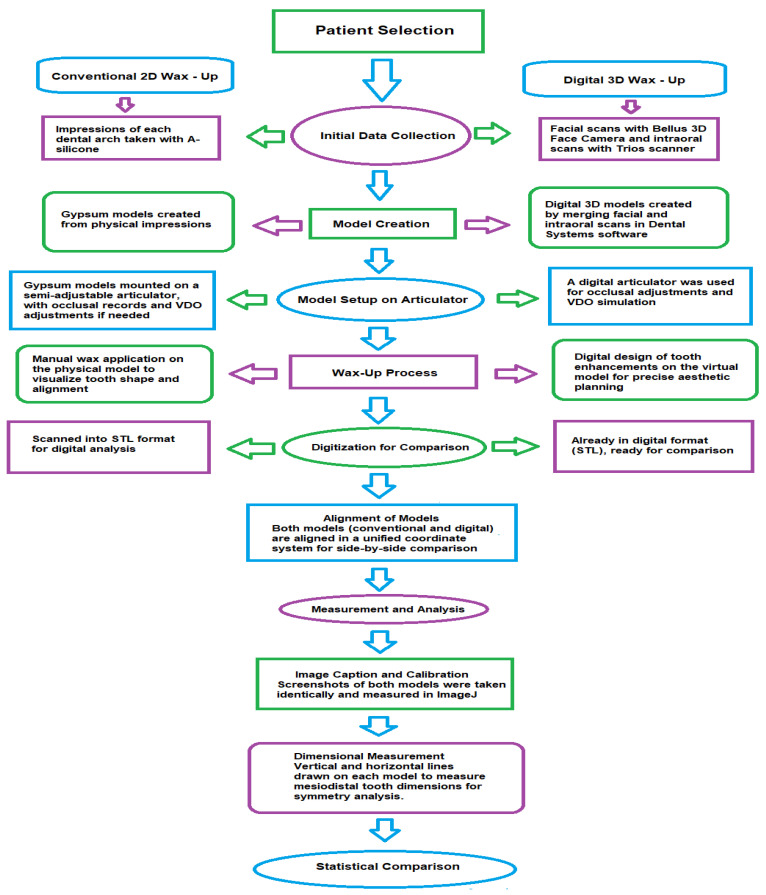
Step-by-step comparison of conventional and digital wax-up methods.

For each patient, two distinct types of wax-ups were executed to facilitate a comparative analysis: a conventional wax-up and a facially driven digital wax-up (Figure 1).

**Figure 2:**
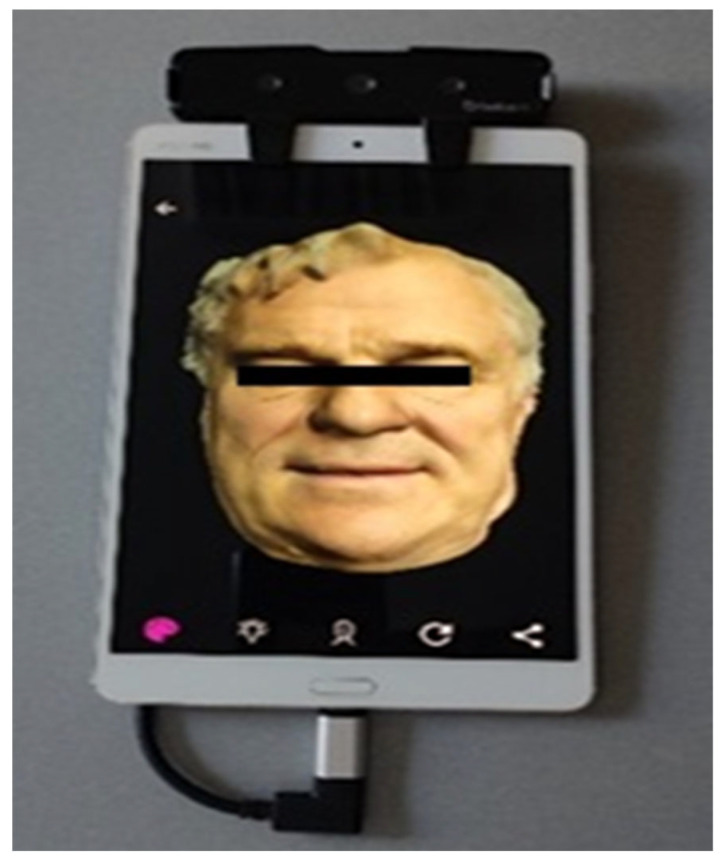
Face Camera Bellus 3D connected with tablet Huawei MediaPad M3 BTV-W09 (Huawei, Shenzhen, China).

The process commenced with acquiring facial scans in two specific conditions: one with the patient displaying a wide smile and another with the lips retracted. These scans were captured using the Face Camera Bellus 3D (Bellus3D, Campbell, CA, USA) and the Huawei MediaPad M3 BTV-W09 tablet (Huawei, Shenzhen, China) (Figure 2). The Face Camera Bellus 3D is renowned for its precision in capturing detailed facial features, while the Huawei MediaPad M3 BTV-W09 tablet provides a reliable platform for integrating and processing scanned data.

The conventional wax-up was created using traditional techniques involving physical materials and manual adjustments. In contrast, the facially driven digital wax-up utilized digital facial scans to simulate and plan aesthetic modifications with enhanced precision. The digital approach enabled the visualization of potential outcomes in a virtual environment before any physical alterations were made.

A conventional diagnostic wax-up was created for each patient following the conventional impression of both dental arches, with the gypsum models mounted on a semi-adjustable articulator. An occlusal record was taken with polyvinyl siloxane when necessary. If there was a need to increase the vertical dimension of occlusion (VDO), adjustments were made in millimeters on the articulator. For the digital protocol, an occlusal scan was performed using intraoral scanning, and a digital articulator was employed to adjust the VDO in millimeters when indicated, as available in Dental Systems by 3Shape (Copenhagen, Denmark).

Conventional impressions of each dental arch were taken using A-silicone, and two sets of models were created for each patient: one set to document the pretreatment condition and another for the analog diagnostic waxing. To facilitate the digital analysis, the intraoral dental structures were captured using the intraoral scanner Trios (3Shape, Copenhagen, Denmark) or through laboratory scanning of the diagnostic casts using the 3Shape E2 scanner.

**Figure 3:**
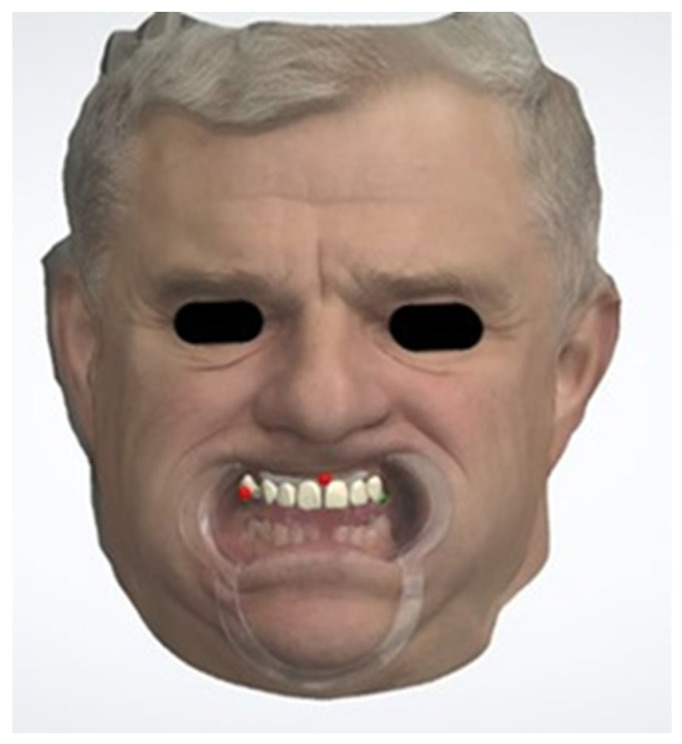
Superimposition of facial and intraoral scans for 3D virtual patient creation in 3D CAD software (Dental Systems; 3Shape, Copenhagen, Denmark): 3D digital reproduction of the scanned patient (Patient ID No.3).

Once the patient’s facial and dental data were digitized, a virtual 3D patient model was generated. The process involved superimposing the facial scan provided in “PLY” format, with the dentition data provided in “STL” format, using advanced 3D CAD software, Version 2023 (Dental Systems; 3Shape, Copenhagen, Denmark). These datasets were fused through a surface-matching algorithm known as Iterative Closest Point (ICP) [31]. Subsequently, a facially driven digital 3D smile design was created, allowing for precise visualization and planning of the aesthetic modifications (Figure 3). This process facilitated a detailed and accurate representation of the intended smile design, integrating facial and dental data to enhance treatment planning and predictability.

In addition to the digital diagnostic wax-up, a conventional diagnostic wax-up was created for each patient. This conventional wax-up was then digitized through laboratory scanning to facilitate comparison in our study. The digitized analog wax pattern was represented as an “STL” file and was incorporated into the digital diagnostic wax-up process. This allowed for a comprehensive analysis by aligning the digital representation of the conventional wax-up with that of the digital diagnostic wax-up.

**Figure 4:**
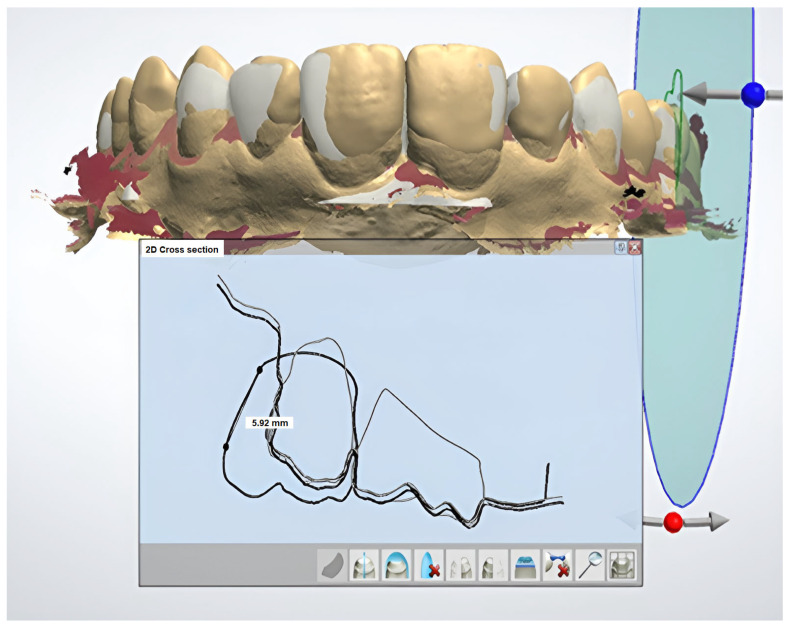
Maximum alignment of the alternative wax-ups. A real size measurement from point to point using the tools in “2D cross section”. (This measurement is used for calibration of the dimensions of the saved images (Patient ID No.4).

The “STL” files from conventional and digital diagnostic wax-ups were aligned within a single coordinate system to ensure accurate comparison. This alignment process involved integrating both data sets into a unified framework, allowing for a direct and precise comparison of the two methods. Figure 4 illustrates this alignment process, showcasing how the images of the conventional and digital diagnostic wax-ups were harmonized for evaluation. This methodological approach enabled a detailed assessment of the similarities and differences between the conventional and digital wax-up techniques.

**Figure 5:**
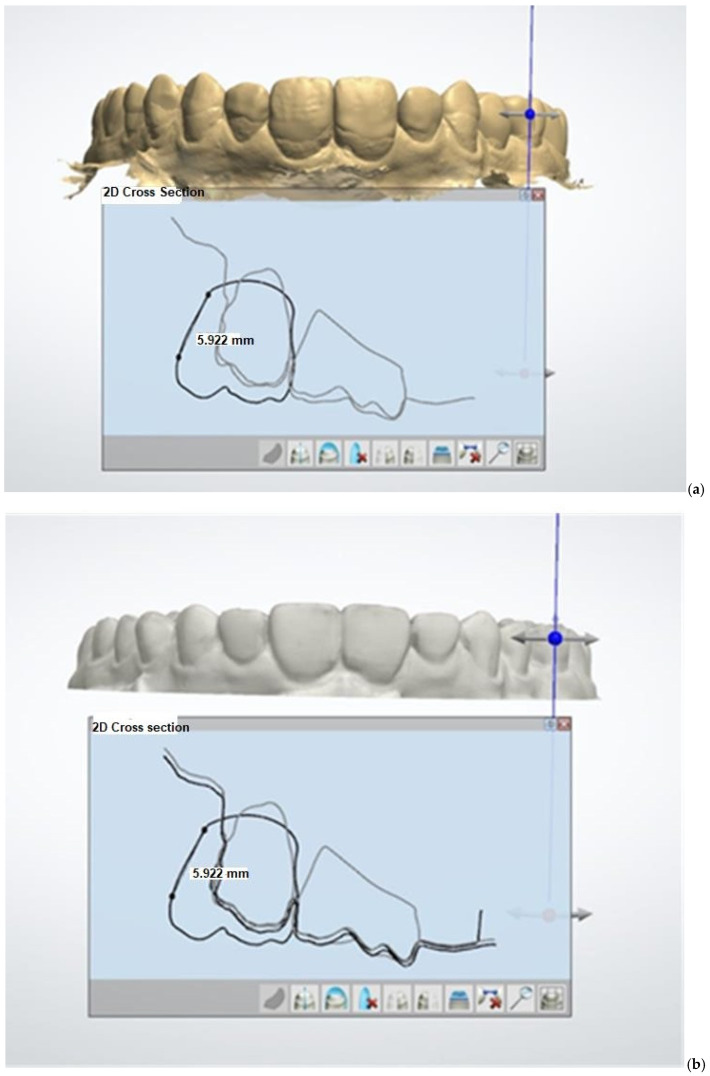
Screenshots of (**a**) digital wax-up and (**b**) digitized analog wax pattern captured in the same position and magnification.

The images of the superimposed models in one coordinate system were saved as screenshots with one and the same magnification and in the same position (frontal view- centered on the central incisors) to make the measurements on their details comparable. (Figure 4). The screenshots from the two alternative models were uploaded to ImageJ, an open-access software for scientific image processing and analysis [30]. The dimensions of the saved images were calibrated. A real size was measured from point to point using the tools in “2D cross section”. An image of this point-to-point distance for calibration was saved in the same screenshot with each of the two models—digital wax-up and digitized conventional one (Figure 5). A calibration of the dimensions of the saved images was performed with the instrument “Set scale” from the ImageJ software (Figure 4).

**Figure 6:**
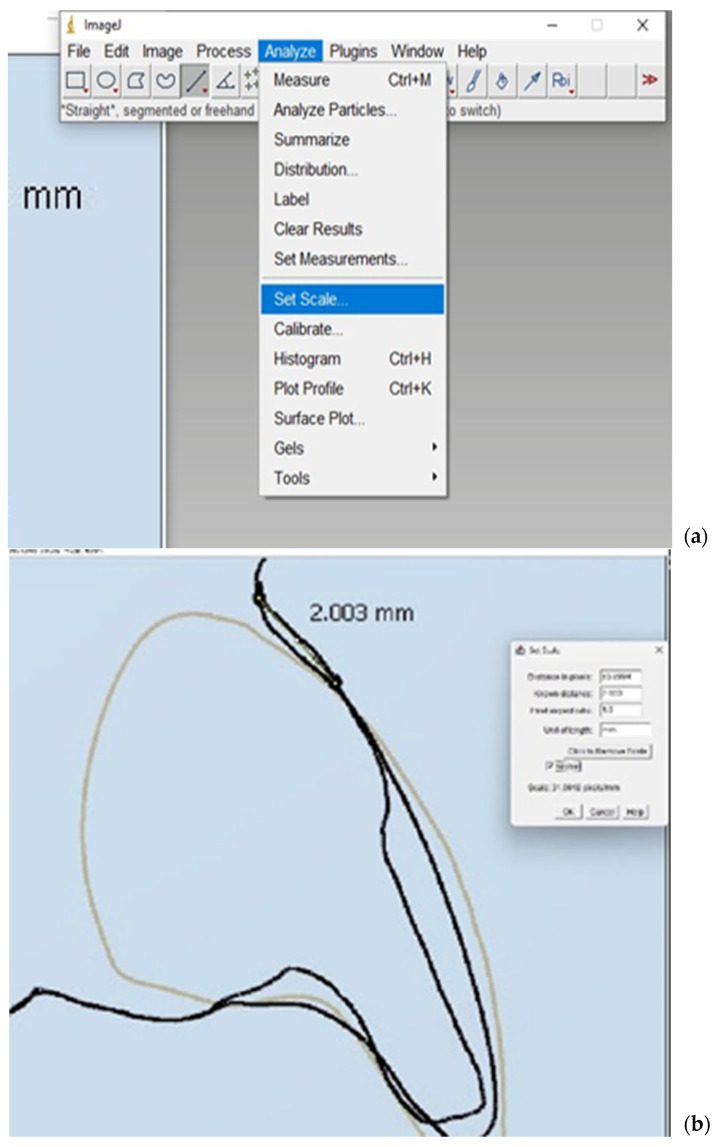
Calibration instrument in ImageJ: (**a**) tools and properties for calibration; (**b**) steps of the calibration process.

Calibration of the dimensions in the saved images was carried out using the “Set scale” tool in ImageJ, which allows for precise adjustment and verification of measurements based on the calibration image (Figure 6). This process ensured that measurements between the two models were consistent and comparable, enabling a rigorous evaluation of their respective dimensions.

Using the tools available in ImageJ, vertical lines were drawn along the central portion of the vestibular surface of each crown on the uploaded screenshots of both the digital and conventional wax-ups. These vertical lines were positioned in the middle section of each crown to ensure consistency. One millimeter from the incisal edge of each crown, a horizontal line was then drawn perpendicular to the vertical lines. This horizontal line extended from the extreme left to the extreme right contour of the crown, thereby allowing for the measurement of the mesiodistal dimension of each tooth.

**Figure 7:**
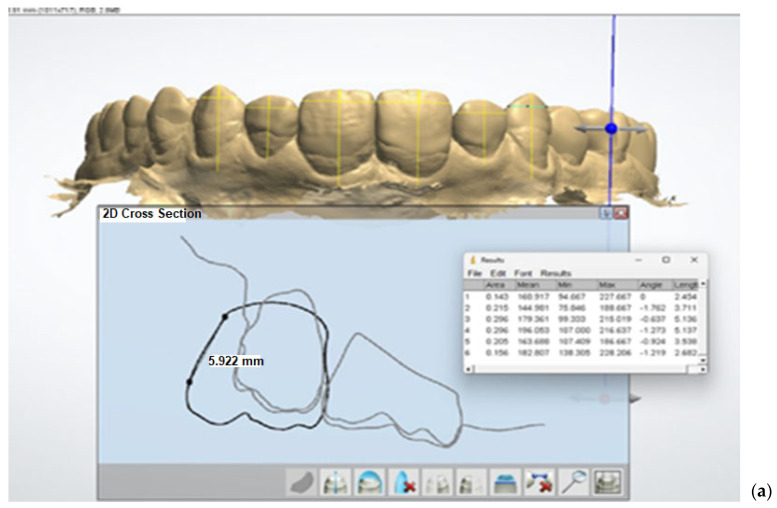

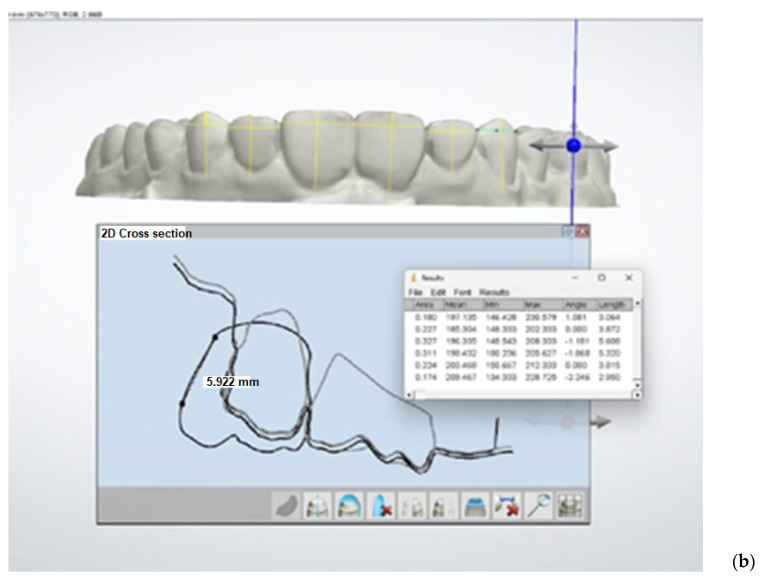
Measurements of mesiodistal dimensions of anterior teeth in ImageJ obtained by (**a**) digital diagnostic waxing and (**b**) conventional wax-up.

The dimensions measured along these horizontal lines were recorded and saved for further statistical analysis. This approach ensured that the mesiodistal dimensions of the teeth were accurately captured and compared across both wax-up methods. The values obtained from these measurements were used to evaluate the symmetry and dimensional accuracy of the different wax-up techniques, providing critical data for the subsequent statistical analysis (Figure 7). This systematic method allowed for a precise assessment of the aesthetic outcomes of both digital and conventional diagnostic wax-ups.

The primary data collected were coded and entered into Microsoft Excel spreadsheets for subsequent analysis. The tooth width values were initially divided into left and right sides. In the first step, for each model, a subtraction (right/left) is performed to assess the symmetry difference for the given model. In the second step, the differences between the digital and conventional models are compared, and absolute values are always taken after each subtraction operation. Consequently, the dataset of 50 individual teeth was consolidated into 25 pairs. A *t*-test was conducted to assess the differences in the symmetry between the digital and conventional wax-up methods. This study compared the average symmetry differences obtained from the digital and conventional wax-ups.

## 3. Results

**Figure 8:**
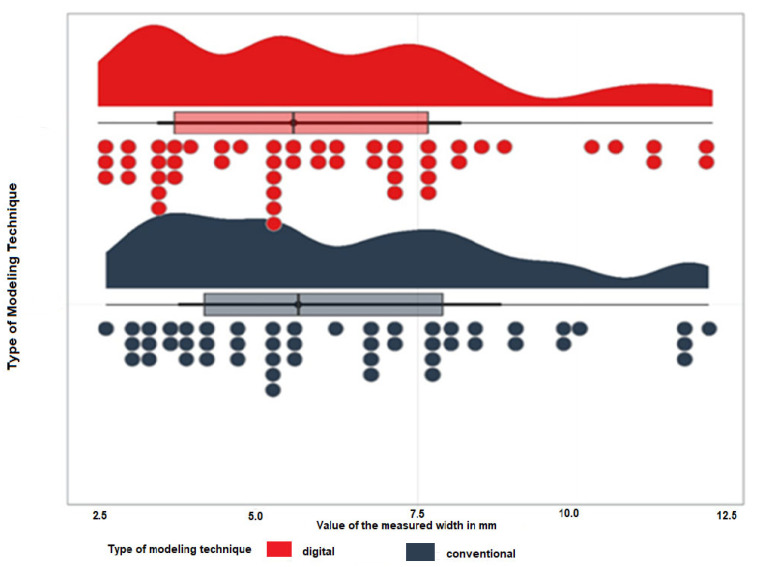
Values of the measured MD diameters in mm.

A total of 50 teeth were evaluated, which were modeled using both the traditional wax-up technique and the face-guided digital approach. The average measurements for each group are illustrated in Figure 8 and detailed in Table 2 and Table 3.

No significant difference was found in the symmetry results between the two modeling methods. This outcome is consistent with the findings of Abduo et al., who investigated the morphological symmetry of upper frontal teeth using conventional and digital diagnostic modeling techniques [15].

For the purpose of our investigation, the difference between the eponymous teeth in the first and second quadrants per patient was calculated. The latter was performed for both digital and conventional models. Twenty-five pairs of teeth values were compared. Mean values are presented in Table 4 and Figure 8.

**Figure 9:**
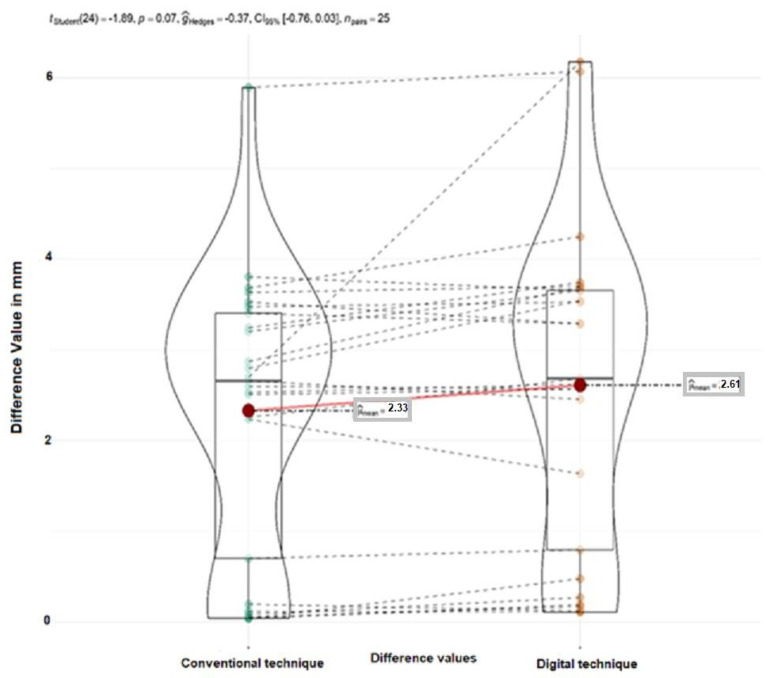
The differences were obtained by subtracting the values for “left-right” variables for the two modeling groups—conventional and digital.

A paired Student’s *t*-test was employed to compare the differences in the symmetry between digitally and conventionally modeled frontal teeth. The outcomes of this analysis are illustrated in Figure 9. This statistical approach enabled a detailed examination of any potential variations in symmetry achieved by the two modeling techniques (Figure 9).

The test results indicated no statistically significant differences when comparing the “right-left” variable values between the conventional and digital modeling groups. Specifically, the statistical analysis yielded a t-value of −1.89 with a *p*-value of 0.07, suggesting that the observed differences in symmetry between the two methods were not significant. This outcome implies that both modeling techniques achieved comparable symmetry results.

## 4. Discussion

This study aimed to compare the accuracy and effectiveness of digital versus conventional wax-ups in reshaping tooth morphology, focusing on linear dimensions within a single horizontal plane. The accepted null hypothesis is that no significant difference in bilateral symmetry exists between digital and conventional diagnostic wax-ups for frontal dentition. The study confirmed that both methods achieved similar symmetry in the transverse plane of the mirroring teeth, indicating that the type of wax-up does not significantly affect the aesthetic outcomes.

Both digital and conventional wax-ups involve three-dimensional reshaping of tooth morphology. However, this study compares linear dimensions within a single horizontal plane. Despite this approach, the findings indicate a high similarity between the results from the two planning methods. These results align with the conclusions of Abduo et al., who also observed similar outcomes in the morphological symmetry of upper frontal teeth using conventional and digital diagnostic modeling techniques [15].

The outcomes of the mock-ups could be influenced by the experience and skill level of the dental specialists involved. Highly skilled professionals carried out all diagnostic wax-ups, which might impact the results. Moreover, digital diagnostic design is a relatively new approach with an ongoing learning curve, as highlighted by Taut et al. [32]. The evolving nature of digital techniques could influence their effectiveness compared to traditional methods.

Moreover, Chrisnoiu et al. highlight that achieving optimal aesthetic results, particularly when modifying the morphology of anterior teeth, relies on a well-structured treatment plan and predictable outcomes [29]. Adaptation to new techniques is crucial for achieving the best possible results. This insight helps explain why their research revealed a patient preference for conventional wax-ups over digital ones, indicating that while digital methods are promising, the conventional approach might still offer more reliable and satisfactory outcomes [32]. This preference reflects the ongoing adaptation and refinement required for digital technologies to match or exceed the effectiveness of traditional techniques [33,34,35,36].

The adoption of digital technology in aesthetic dentistry provides significant clinical advantages, particularly in enhancing efficiency and reducing patient chair time. Digital diagnostic wax-ups and 3D imaging allow for precise visualization of treatment outcomes, streamlining workflows and enabling quicker modifications. These methods improve integration with patient management systems, fostering better communication with patients and potentially decreasing appointment cancellations, leading to higher satisfaction [37]. Additionally, CAD/CAM systems facilitate faster design and fabrication of restorations, minimizing the need for multiple visits. However, transitioning to digital methods involves a learning curve, necessitating ongoing education for practitioners to fully leverage these benefits as technologies continue to evolve [38].

Comparative studies have explored the reliability and efficacy of digital versus conventional wax-ups. For instance, research by Smith et al. [39] demonstrated that digital wax-ups offer similar precision to traditional methods, particularly regarding symmetry and dimensional accuracy. Their findings, however, noted that digital methods might involve a steeper learning curve but could provide superior efficiency and reproducibility once mastered. Similarly, Jones et al. [40] conducted a study focusing on the clinical outcomes of digital versus conventional wax-ups. They found that while both methods yielded similar aesthetic results, digital wax-ups were advantageous in terms of integration with other digital tools and patient management systems. Their study highlighted that digital techniques could streamline workflows and enhance collaboration among dental professionals. In contrast, Patel et al. [41] observed some limitations with digital wax-ups, particularly in the initial phases of adoption. They noted that while digital methods are highly accurate, they require significant adjustments and familiarity with new software and hardware. This study underscored that conventional wax-ups might still hold an edge in terms of immediate reliability and ease of use, particularly in cases where rapid adjustments are needed.

Compared to these studies, the current research underscores the reliability of digital diagnostic wax-ups as equivalent to conventional methods, aligning with the findings of Smith et al. and Jones et al. [42,43]. However, it also supports the notion that digital techniques, while promising, are still evolving and may face challenges similar to those noted by Patel et al. [44]. As digital technologies advance and become more widely adopted, they are likely to further bridge any gaps between digital and conventional approaches, offering enhanced precision and efficiency in dental diagnostics [44,45,46].

An interesting aesthetic aspect involves the natural beauty of a smile, which is not always perfectly symmetric. This quality is exemplified by figures such as Tom Cruise, whose slightly asymmetrical smile contributes to its unique charm and appeal [47]. While achieving near-perfect symmetry in tooth morphology is often the goal of both digital and conventional wax-ups, it is important to consider that a beautiful smile can still thrive with subtle asymmetries, which can add character and authenticity. This aspect underscores the value of tools like digital smile design (DSD) and wax-ups, allowing for precise, personalized planning. Through digital methods, clinicians can simulate various smile designs, helping balance symmetrical and asymmetrical elements based on the patient’s features [48]. Similarly, traditional wax-ups offer hands-on adjustments that can be guided by artistic intuition. These tools thus provide valuable flexibility, enabling clinicians to achieve aesthetic results that are accurate and align with each patient’s natural beauty and individuality.

Additionally, 3D planning in smile design relies substantially on both clinical expertise and the growing capabilities of artificial intelligence (AI). Integrating AI into the process brings the advantage of potential standardization and predictive support, helping clinicians visualize and assess possible treatment outcomes [49]. However, AI’s utility is inherently tied to the quality of its training data and the clinician’s ability to interpret and apply its suggestions effectively. Ultimately, the success or failure of any treatment is grounded in the practitioner’s skill, judgment, and experience. While digital tools and AI can greatly aid in enhancing precision, they are not substitutes for the hands-on expertise and critical decision-making required to plan and execute optimal patient outcomes [50].

Achieving dental symmetry is particularly challenging in patients with tooth loss or congenital deformities like clefts, which often result in irregularities in tooth size and alignment. As noted by Freitas et al., these conditions require a nuanced smile design approach that prioritizes harmonious balance over strict symmetry [51]. Digital Smile Design (DSD) tools enable clinicians to simulate aesthetic outcomes and create tailored treatment plans that accommodate these complexities. Ultimately, it is essential to appreciate how asymmetries can enhance a smile’s character, reinforcing the notion that beauty lies in individuality.

The integration of CAD/CAM technology in dental treatment planning significantly enhances the durability of prosthetic restorations. These systems ensure precise fit and design, which leads to improved mechanical properties and greater resistance to damage. This advancement not only increases patient satisfaction and comfort but also reduces the need for repairs, streamlining the overall workflow in dental practices [52,53].

One significant limitation of this study is the potential bias stemming from the technicians’ varying experience levels who performed the wax-ups. Since all technicians were highly experienced professionals, the outcomes may not accurately represent the variability that could occur with less experienced practitioners, possibly favoring the conventional method. Additionally, the focus on linear dimensions within a single horizontal plane limits the analysis to a two-dimensional viewpoint, which may fail to capture the complexities involved in three-dimensional reshaping. The relatively small sample size also constrains the generalizability of the findings, indicating a need for further research involving a larger participant group to confirm these results. Future investigations should include technicians with diverse skill levels to determine how experience influences the effectiveness of both digital and conventional wax-ups. Incorporating three-dimensional assessments into the analysis would offer a more thorough understanding of the distinctions between the two methods. Furthermore, longitudinal studies are crucial to assess the long-term performance and clinical outcomes of both techniques, along with patient feedback to provide insights into real-world applications and levels of satisfaction.

## 5. Conclusions

Our findings confirmed the null hypothesis, demonstrating that both methods produced comparable symmetry in the transverse plane of the corresponding teeth, indicating that the type of wax-up does not significantly impact aesthetic outcomes. Digital modeling has proven to be an effective tool for analyzing and planning prosthetic reconstructions, closely aligning with traditional wax modeling techniques. By enhancing visualization and symmetry assessment, digital modeling empowers clinicians to make informed decisions tailored to each patient’s unique background. As technology advances and clinician expertise develops, there is significant potential for further exploration of the relationship between upbringing and physical attributes. Ultimately, this study aims to provide valuable insights into the effectiveness and accuracy of digital technologies in achieving aesthetically pleasing and symmetrical outcomes, which could influence future practices in dental prosthodontics and smile design.

## Figures and Tables

**Table 1:** Patients participating in the study with teeth modeled using digital and conventional wax-up techniques.

Patient ID	Gender	Teeth Waxed Up Digitally	Teeth Waxed Up Conventionally
No.1	female	13, 12, 11, 21, 22, 23	13, 12, 11, 21, 22, 23
No.2	female	13, 12, 11, 21, 22, 23	13, 12, 11, 21, 22, 23
No.3	male	13, 12, 11, 21, 22, 23	13, 12, 11, 21, 22, 23
No.4	male	12, 11, 21, 22	12, 11, 21, 22
No.5	female	11 (natural tooth 21 was measured for comparing symmetry)	11 (natural tooth 21 was measured for comparing symmetry)
No.6	female	12, 11, 21, 22	12, 11, 21, 22
No.7	male	12, 11, 21, 22	12, 11, 21, 22
No.8	female	13, 12, 11, 21, 22, 23	13, 12, 11, 21, 22, 23
No.9	male	13, 12, 11, 21, 22, 23	13, 12, 11, 21, 22, 23
No.10	female	13, 12, 11, 21, 22, 23	13, 12, 11, 21, 22, 23

**Table 2:** Mean values of the two alternative wax-up techniques—digital/conventional.

Type of Wax-Up Technique	n	Mean	SD
Conventional	50	6.32	2.62
Digital	50	6.05	2.62

**Table 3:** Descriptive analysis of the obtained data.

Type of Wax-Up Technique	n	Mean Values of the Difference (Left/Right)	Confidence Interval (95%)	Standard Deviation (SD)	Overall Mean	Overall SD
			Lower Bound (LB)	Upper Bound (UB)		
Conventional	25	2.33	1.69	2.96	6.32	2.62
Digital	25	2.61	1.88	3.33	6.05	2.62

**Table 4:** Mean values of the difference between the eponymous teeth in the I and II quadrants.

Type of Wax-Up Technique	n	Mean Values of the Difference (Left/Right)	CI (95%)		SD
			LB	UB	
Conventional	25	2.33	1.69	2.96	1.55
Digital	25	2.61	1.88	3.33	1.76

## Data Availability

The data presented in the study are available upon request from the authors.
